# Two-year longitudinal study of *Eimeria uekii* and *Eimeria raichoi* oocyst shedding in Japanese rock ptarmigans (*Lagopus muta japonica*)

**DOI:** 10.1016/j.ijppaw.2025.101088

**Published:** 2025-05-27

**Authors:** Nami Masakane, Mei Harafuji, Yuki Arakawa, Tatsuhiko Yamakami, Naoya Tamura, Sayaka Tsuchida, Atsushi Kobayashi, Tomoyuki Shibahara, Hiroshi Nakamura, Kazumi Sasai, Kazunari Ushida, Makoto Matsubayashi

**Affiliations:** a Graduate School of Veterinary Medical Sciences, Osaka Metropolitan University, Osaka, 598-8531, Japan; b Nasu Animal Kingdom, Tochigi, 329-3223, Japan; c Nagano Chausuyama Zoo, Nagano, 388-8016, Japan; d College of Bioscience and Biotechnology, Chubu University, Aichi, 487-8501, Japan; e Nagano Environmental Conservation Research Institute, Natural Environment Division, Biodiversity Section, Nagano, 381-0075, Japan; f Division of Hygiene Management Research, National Institute of Animal Health, National Agriculture and Food Research Organization, Ibaraki, 305-0856, Japan; g General Foundation Hiroshi Nakamura International Institute for Ornithology, Nakagosho, Nagano, 380-0934, Japan

**Keywords:** *Eimeria*, Japanese Alps, Japanese rock ptarmigan, Oocyst

## Abstract

The Japanese rock ptarmigan (*Lagopus muta japonica*) is a cold-adapted bird that inhabits only alpine areas of Japan. The birds have been reported to be highly infected with two *Eimeria* species; however, these *Eimeria* species including long-term infection dynamics have not yet been well documented. Since surveys requiring access into the mountain areas of the Japanese Alps are prohibited from November to April (winter), the prevalences of eimerian parasites and details on their transmission routes among the wild birds in the habitat in winter remain unknown. As part of *ex situ* conservation programs for Japanese rock ptarmigans, two families, including the female parent and chicks, were transported to two zoos for breeding in 2021. To examine fluctuations in the infection status of *Eimeria* species in Japanese rock ptarmigans, we conducted a 2-year longitudinal study of oocyst shedding. We found that all examined birds were continuously infected with two *Eimeria* species, although oocysts were sometimes not detected in feces. On average, the oocysts per gram (OPG) values were <100; however, those of female parent birds that successfully laid fertilized eggs and/or cared their chicks increased by more than 100 to 1000 before or when the chicks hatched. Subsequently, all of the chicks became infected with the *Eimeria* species. The OPG values did not drastically change in the female birds that were not paired for breeding or had laid unfertilized eggs, and in the male birds. Although further studies are needed, our findings indicated that the parasites are transmitted from female parents to chicks after hatching, and that the birds may be continuously infected, showing low OPG levels.

## Highlights

• A 2-year longitudinal study of eimerian oocyst shedding were conducted in Japanese rock ptarmigans.
• All examined birds were continuously infected with two *Eimeria* spp. at low OPG levels.
• OPGs of only female parent birds that successfully bred increased in the breeding season.

## Graphical abstract

**Figure:**
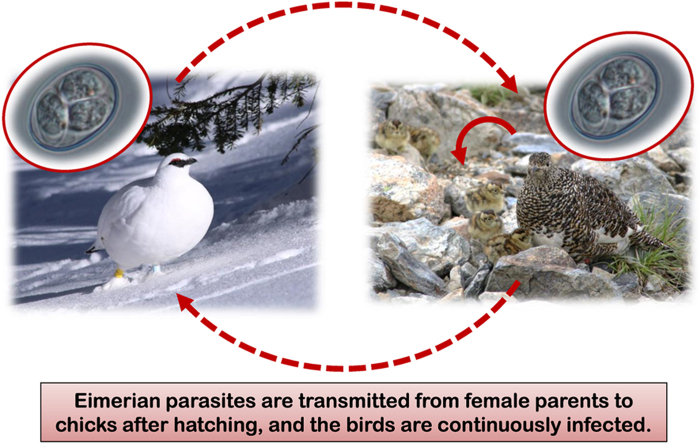

## 1 Introduction

The Japanese rock ptarmigan, *Lagopus muta japonica*, is one of 31 subspecies belonging to the family Phasianidae, order Galliformes (Avibase, 2024). The bird inhabits only alpine areas of the main island of Japan, which is the southernmost habitat of rock ptarmigans, and is isolated from other subspecies (Birds of the World https://birdsoftheworld.org/bow/home). Japanese rock ptarmigans mate within their territories in April (spring), and lay and incubate eggs between May to July (Nakamura, 2007). After their chicks hatch around July (summer), the pairs and their territories are broken up while the female parent bird takes care of the chicks as a family until October (autumn). After the chicks leave their parents, they live alone or in a group with adult birds. Due to a decrease in the number of the birds, which fell to approximately 1700 in the 2000s, the Japanese rock ptarmigan is currently listed as an endangered species on the Fourth Red List of Threatened Species of the Ministry of the Environment of Japan (Ueda et al., 2018; Ministry of the Environment of Japan, 2020). Therefore, both *in situ* and *ex situ* national conservation programs for Japanese rock ptarmigans are being conducted (Ministry of Environment Japan, 2012).

*Eimeria* parasites are alveolates of the phylum Apicomplexa. They are highly host-specific, and have been detected in a wide range of animals, such as livestock and wild mammals, including birds (Cowper et al., 2012). Generally, after the oral ingestion of mature oocysts, sporozoites are released, and they invade mainly the intestinal mucosa of the host. After asexual and sexual development, newly produced oocysts are shed in the feces during the patent period, the timing of which differs among *Eimeria* spp. The shed oocysts subsequently sporulate into the infective mature form when the temperature is appropriate (approximately 25 °C–27 °C for most species), and they can survive in the environment for several months. Wild Japanese rock ptarmigans have been reported to be highly infected by two *Eimeria* species, *i.e., Eimeria uekii* Ishihara et al., 2006 and *Eimeria raichoi* Matsubayashi et al., 2018a, and the prevalences range from 36.1 % to 84.8 % (Kamimura and Kodama, 1981; Ishihara et al., 2006; Matsubayashi et al., 2018a, 2018b). The pathogenicity of the *Eimeria* species in Japanese rock ptarmigans has not yet been fully clarified. However, results from studies of experimental infections in Svalbard rock ptarmigans (*Lagopus muta hyperboreus*) as a subspecies of Japanese rock ptarmigans have suggested that high-dose inoculation of the oocysts derived from Japanese rock ptarmigans could induce clinical symptoms, such as diarrhea and reduced weight gain, that resulted in the death of some birds (Matsubayashi et al., 2023).

One reason for the lack of data on the two *Eimeria* species that infect Japanese rock ptarmigans, including data on their pathogenicity, is that surveys requiring access into the mountain areas of the Japanese Alps are prohibited from November to April (winter). Thus, the prevalence of eimerian parasites and details on their transmission routes among the wild birds in the habitat during winter remain unknown. Previously, it was suggested that the oocysts of eimerian parasites infecting Japanese rock ptarmigans could mature at relatively lower temperatures (approximately 15 °C–20 °C), enabling them to survive longer (for approximately 6 months) outside of the hosts (Matsubayashi et al., 2018b, 2021). In another study, it was reported that freezing at a temperature less than −10 °C could kill the oocysts of *Eimeria* species that infect cattle and sheep (Landers, 1953; Lassen and Seppä-Lassila, 2014). The temperature at the Japanese Alps may reach less than −10 °C, so it is possible that the oocysts may not remain infective in these areas. Additionally, another study reported that no oocysts could be detected from soil samples collected from the habitats in the Japanese Alps (Matsubayashi et al., 2021). Therefore, the dynamics, including the transmission routes, of these oocysts that are shed from the hosts during the patent period in alpine areas remain unknown.

As part of *ex situ* conservation and breeding programs for Japanese rock ptarmigans, two families, including the female parent and chicks, were transported to two zoos for breeding in 2021. To examine fluctuations in the infection status of *Eimeria* species in Japanese rock ptarmigans, we conducted a 2-year longitudinal study of oocyst shedding.

## 2 Materials and methods

### 2.1 Examined birds

**Fig. 1:**
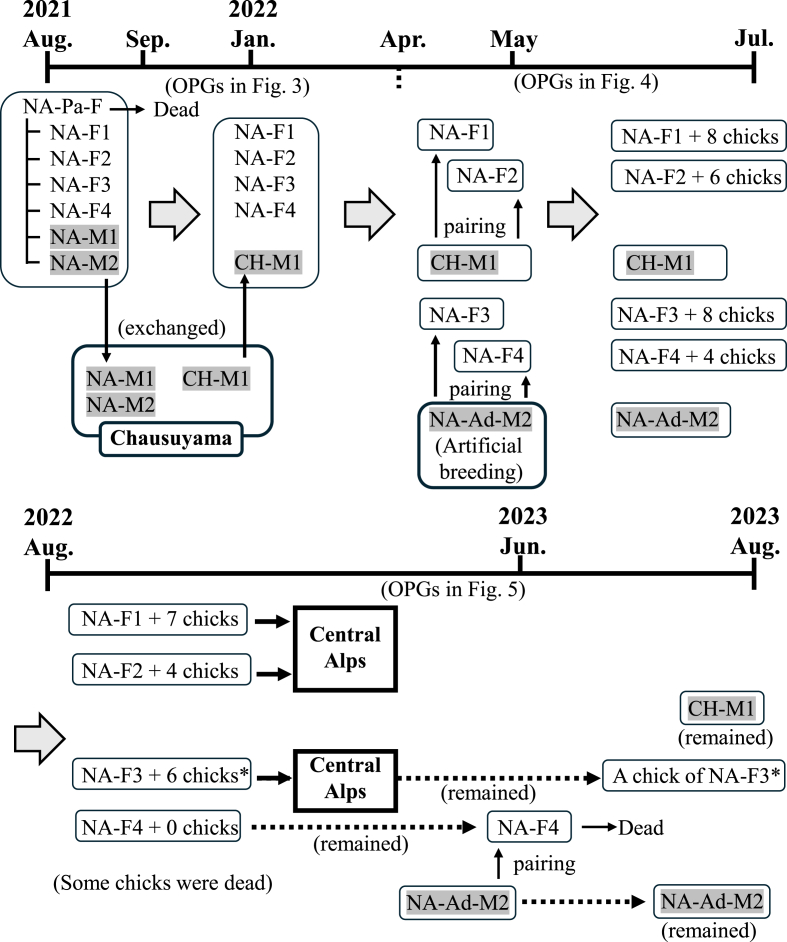
Summary of the breeding of Family A at Nasu Animal Kingdom from August 2021 to August 2023. Each frame indicates the same raising. Male birds are indicated by gray shading. NA-Pa-F: parent female bird of Family A. NA-F1 to F4 (female) and NA-M1 and M2 (male): chicks of NA-Pa-F. CH-M1 (male): a chick from Family B that was moved from Nagano Chausuyama Zoo. NA-Ad-M2: male adult bird that was artificially bred at Nasu Animal (approximately six-year-old). Some birds were moved to their natural habitat in the Central Alps in 2022 and 2023. One chick (∗) among six hatched chicks of NA-F3 remained.

**Fig. 2:**
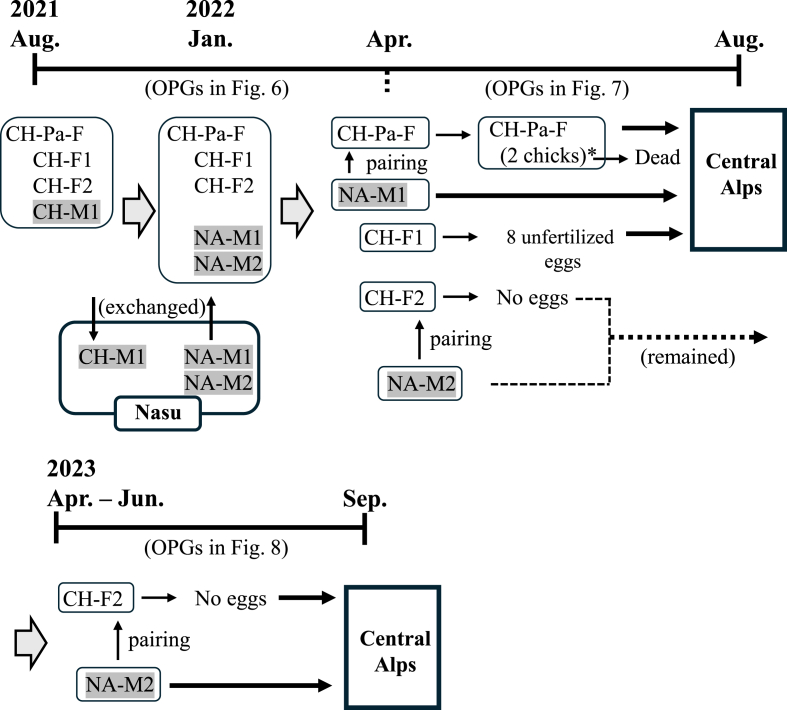
Summary of the breeding of Family B at Nagano Chausuyama Zoo from August 2021 to August 2023. Each frame indicates the same raising. Male birds are indicated by gray shading. CH-Pa-F: parent female bird of Family B. CH-F1 to F2 (female) and CH-M1 (male): chicks of NA-Pa-F. NA-M1 and M2 (male): a chick from Family A that was moved from Nasu Animal Kingdom. Some birds were moved to their natural habitat in the Central Alps in 2022 and 2023.

In July 2021, two families of Japanese rock ptarmigans (Family A: one female parent bird and four female and two male chicks as shown in Fig. 1; Family B: one female parent bird and two female and one male chicks as shown in Fig. 2) were used, which were inhabited at Mt. Komagatake (35°79′N, 137°80′E) in Nagano Prefecture in the Central Japanese Alps. In August 2021, Family A was moved to Nasu Animal Kingdom in Tochigi, Japan, and Family B was moved to Nagano Chausuyama Zoo in Nagano, Japan. Subsequently, for breeding, two male chicks (NA-M1 and NA-M2) from Family A were exchanged with one chick from Family B (CH-M1) in January 2022; breeding was attempted after March to April 2022 as shown in Fig. 1, Fig. 2. After breeding, some of the birds were sent to the Central Japanese Alps in August 2022 and September 2023. All birds were cared for according to the protocols of the conservation and breeding projects for Japanese rock ptarmigans established by Ministry of the Environment.

The birds (or families) were kept in separate isolated rooms at night and released into free-range areas during the day. The feces were removed and the floor was washed with water and treated with disinfectants every day (cationic detergents; Scientific Feed Laboratory, Tokyo, Japan or Meiji Animal Health, Kumamoto, Japan), and the gravel or sand were periodically changed. In the free-range areas, the feces and the sand around the feces were removed every day, and treated with disinfectants once a month.

### 2.2 Fecal samples

Fresh fecal samples of the birds were collected on 1–7 days each week during July and August, and on 2–4 days each month from September to June. While parents cared for their chicks, the feces of the chicks were randomly collected and mixed. Fecal samples were stored at 4 °C, transported to the laboratory, and examined as described below.

### 2.3 Examinations of *Eimeria* parasites

To detect eimerian parasites, the sugar flotation centrifuge method was performed as previously described (Ekawasti et al., 2019; Matsubayashi et al., 2018b). In brief, 1 g of the fecal samples was diluted with tap water and filtrated with a steel mesh or gauze, and sugar solution with a specific gravity of 1.2 was added. The floated parasites were transferred onto a glass slide, and the entire smear was examined under light microscopy. The species of the oocysts (*E. uekii* or *E. raichoi*) were morphologically identified under a microscope (E200, Nikon, Tokyo, Japan) (Matsubayashi et al., 2018a), and the oocysts per gram (OPG) value of the *Eimeria* species was calculated (Takano et al., 2024).

## 3 Results

### 3.1 The OPG in family A

**Fig. 3:**
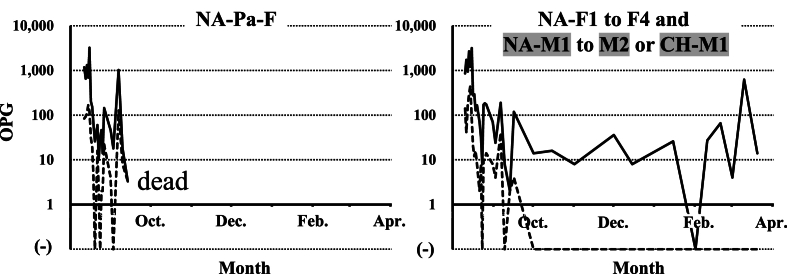
The oocysts per gram (OPG) values (log values of the OPG) for *Eimeria uekii* (solid line) and *Eimeria raichoi* (dashed line) of the female parent bird of Family A (NA-Pa-F) and the chicks (NA-F1 to F4 and NA-M1 to M2 or CH-M1) kept at Nasu Animal Kingdom from August 2021 to April 2022. Male birds are indicated by gray shading. (−) indicates that no oocysts were detected.

**Fig. 4:**
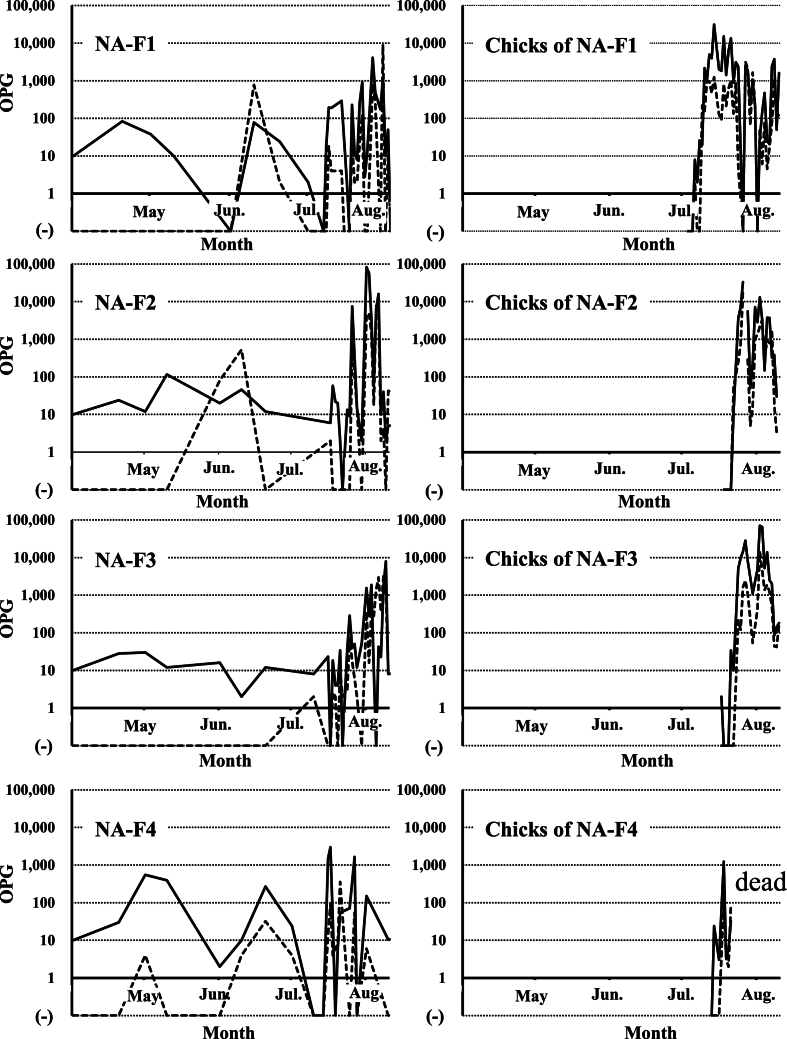
The OPG values (log values of the OPG) for *E. uekii* (solid line) and *E. raichoi* (dashed line) of grown female chicks (NA-F1 to F4) of Family A and their chicks kept at Nasu Animal Kingdom from April to August 2022. (−) indicates that no oocysts were detected.

Fecal examination results showed that all birds in Family A were already infected with the two *Eimeria* species in August 2021 (Fig. 3). The parent bird died in an accident unrelated to infection by *Eimeria* species in September 2021. By fecal examinations, low levels of *E. uekii* oocysts in feces (<100 OPG) were frequently detected, and oocysts of *E. raichoi* were no longer detected by April 2022 (Fig. 3) to June 2022 (NA-F1 to F4 in Fig. 4). On April 2022, all of the grown chicks (NA-F1 to F4) were bred with a male grown chick (CH-M1) and one male adult bird (NA-Ad-M2) that was artificially bred at Nasu Animal Kingdom (approximately six-year-old). The artificially bred adult bird (NA-Ad-M2) was confirmed to be negative with the infection of *Eimeria* species before the breeding. After the breeding and hatching of the chicks, the OPG values of both *E. uekii* and *E. raichoi* in all parent birds increased (>100 OPG) around July and August 2022 (NA-F1 to F4 in Fig. 4). The chicks of four parents were infected by both *Eimeria* species with OPG values of more than 1000 or 10,000. Interestingly, the OPG values of the four parents increased before or at around the same time as when the OPG values of the chicks increased.

**Fig. 5:**
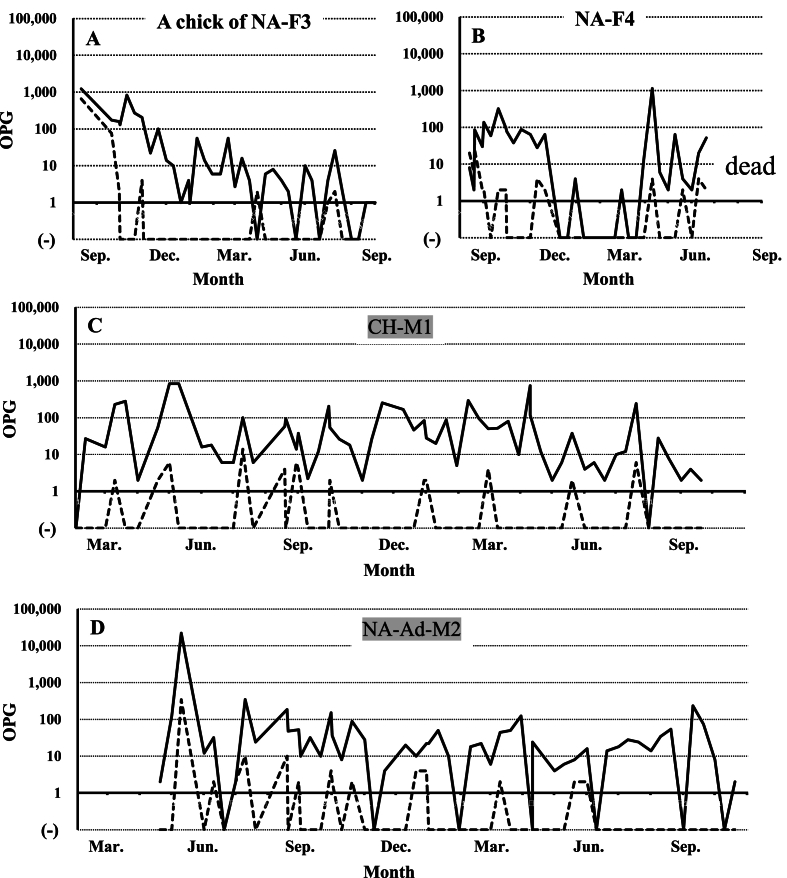
The OPG values (log values of the OPG) for *E. uekii* (solid line) and *E. raichoi* (dashed line) of female birds (a chick of NA-F3, and NA-F4) from August to September 2022, and male birds (CH-M1 and NA-Ad-M2) from February to November 2022. Male birds are indicated by gray shading. (−) indicates that no oocysts were detected.

In August 2022, three parent birds and 16 chicks were moved to their natural habitat in the Central Alps (Fig. 1), and two female birds (one chick of NA-F3, and NA-F4), one male bird (CH-M1), and one artificial breeding male bird (NA-Ad-M2) were kept at Nasu Animal Kingdom. From August 2022 to August 2023, oocysts of the two *Eimeria* species were frequently detected in all four birds (Fig. 5). One of the female birds (one chick of NA-F3) was difficulty walking due to accidental fracture and thus, was not paired for breeding, and the OPG value did not increase for this bird; nonetheless, the infection never completely resolved (Fig. 5A). In the other female bird (NA-F4), relatively low levels of oocysts of both *Eimeria* species were also often found in the feces (Fig. 5B), and the bird died in June 2023 although detailed reasons remained unknown (probably due to ruptured-yolk peritonitis). The male bird (CH-M1) was also chronically infected with the two *Eimeria* species over the 2 years of examinations; the OPG value sometimes reached 100, but did not surpass 1000 (Fig. 5C). The artificially bred male bird (NA-Ad-M2) was also infected with low levels of the parasites (approximately ≤100 OPG) even though the OPG value was more than 10,000 after the initial infection in March 2022 (Fig. 5D). Totally, the maximum OPGs depending on successful breeding and sexes were summarized in Table 1.

**Table 1:** Summary of maximum OPGs for birds reared in Nasu Animal Kingdom.

Categories and birds	Maximum OPG		Notes
	*E. uekii*	*E. raichoi*	
**Successful breeding**			
**Aug. 2021 to Mar. in 2022 after moving to Nasu Animal Kingdom**			
NA-Pa-F	3230.0	168.0	Accidental death
Chicks with NA-Pa-F	3160.0	288.0	NA-F1 to F4 and others
**Apr. to Aug. in 2022**			
NA-F1	4118.0	9032.0	
Chicks with NA-F1	31,368.2	3257.1	One of 8 chicks were dead
NA-F2	82,547.8	7635.7	
Chicks with NA-F2	33,019.1	18,986.0	Two of 6 chicks were dead
NA-F3	7945.2	3312.0	
Chicks with NA-F3	70,036.4	14,072.7	Two of 8 chicks were dead
NA-F4	2988.0	356.0	
Chicks with NA-F4	1231.1	130.6	All of 4 chicks were dead

**No or unsuccess female breeding**			
**Apr. to Aug. in 2023**			
A chick of NA-F3	26.0	2.0	No breeding
NA-F4	1144.0	4.0	Death after pairing

**Male birds**			
**Apr. to Aug. in 2022**			
CH-M1	844.0	14.0	
NA-Ad-M2	348.0 (22,100.0)	10.0 (344.0)	Artificially breeding bird (within one month after parring)
**Apr. to Aug. in 2023**			
CH-M1	242.0	6.0	
NA-Ad-M2	54.0	2.0	Artificially breeding bird

### 3.2 The OPG in family B

**Fig. 6:**
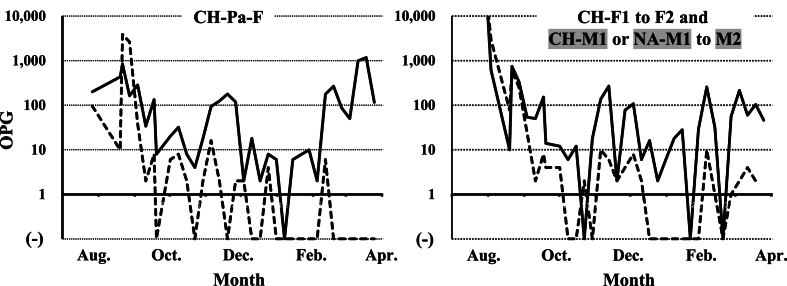
The OPG values (log values of the OPG) for *E. uekii* (solid line) and *E. raichoi* (dashed line) of the female parent bird of Family B (CH-Pa-F) and chicks (CH-F1 to F2, and CH-M1 to M2) kept at Nagano Chausuyama Zoo from August 2021 to April 2022. Male birds are indicated by gray shading. (−) indicates that no oocysts were detected.

**Fig. 7:**
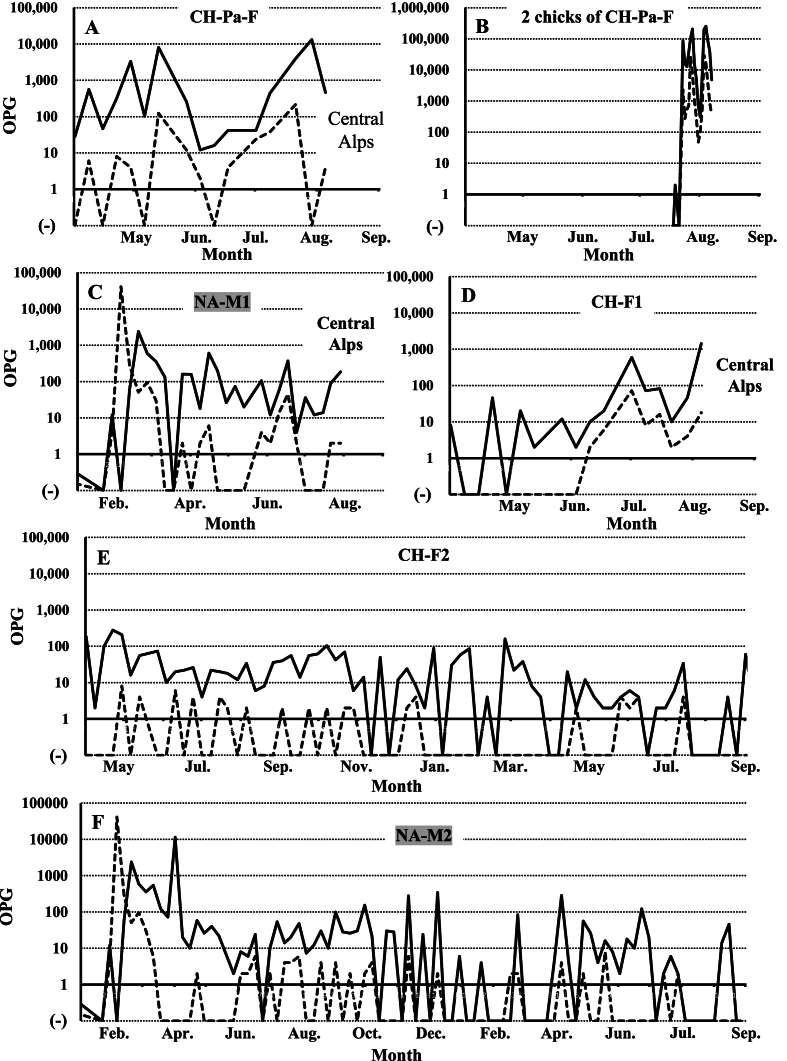
The OPG values (log values of the OPG) for *E. uekii* (solid line) and *E. raichoi* (dashed line) of the female parent bird of Family B (CH-Pa-F), two chicks of CH-Pa-F and grown female chicks at Nagano Chausuyama Zoo (CH-F1 and CH-F2), and grown male chicks moved from Nasu Animal Kingdom (NA-M1 and NA-M2) from April to August 2022 or from January 2022 to September 2023. Male birds are indicated by gray shading. (−) indicates that no oocysts were detected.

In Family B, all chicks and the parent bird were infected with the two *Eimeria* species in August 2021 (Fig. 6). Until April 2022, *E. uekii* and *E. raichoi* oocysts (around 100 OPG) were regularly detected, while they were sometimes not detected. Around April 2022, the parent bird (CH-Pa-F) and one grown female chick (CH-F2) were paired with male birds NA-M1 and NA-M2, respectively (Fig. 2). The parent bird (CH-Pa-F) laid four unfertilized eggs (not shown in Fig. 2), then brooded two fertilized eggs that were moved from another zoo (Toyama Municipal Family Park Zoo, Toyama, Japan). Consequently, two chicks hatched; however, they died within 1 month for unknown reasons. Fecal examinations revealed that the OPG value of the parent bird (CH-Pa-F) increased to more than 100 before the two chicks were hatched (after April 2022), and that it subsequently increased to over 10,000 (Fig. 7A). The two hatched chicks shed high levels of oocysts in feces (>100,000 OPG) (Fig. 7B). The male bird (NA-M1) that was paired with the parent bird (CH-Pa-F) shed oocysts (around 100 OPG) (Fig. 7C). On the other hand, one grown chick (CH-F1) that was not paired for breeding and laid 8 unfertilized eggs shed lower levels of oocysts (<100 OPG), although the value increased more than 100 a few times during the examined period (Fig. 7D).

In the other pair, one grown chick (CH-F2) was bred with a male bird (NA-M2) in 2023 in addition to the attempt in 2022. However, the bird did not lay any eggs. During the examined period, the OPG values of the grown chick (CH-F2) and the male bird (NA-M2) were approximately less than 100 (Fig. 7E and F). In summary, the maximum OPGs depending on successful breeding and sexes were shown in Table 2.

**Table 2:** Summary of maximum OPGs for birds reared in Nagano Chausuyama Zoo.

Categories and birds	Maximum OPG		Notes
	*E. uekii*	*E. raichoi*	
**Successful breeding**			
**Aug. 2021 to Mar. in 2022 after moving to Nagano Chausuyama Zoo**			
CH-Pa-F	1170.0	3884.0	
Chicks with CH-Pa-F	2,268,098.0	336,260.0	NA-F1 to F4
**Apr. to Aug. in 2022**			
CH-Pa-F	13,207.6	216.0	
Chicks with CH-Pa-F	254,718.8	30,267.5	All of 2 chicks were dead

**No or unsuccess female breeding**			
**Apr. to Aug. in 2022**			
CH-F1	1422.0	72.0	8 unfertilized eggs (no pairing)
CH-F2	278.0	8.0	No eggs after pairing
**Apr. to Aug. in 2023**			
CH-F2	60.0	4.0	No eggs after pairing

**Male birds (Apr. 2022 to Aug. 2023)**			
NA-M1	606.0	45.0	
NA-M2	58.0 (11,454.0)	6.0	(unknown transient increasing only on April 1, 2022)
**Male birds (Apr. 2022 to Aug. 2023)**			
NA-M2	284.0	8.0	

## 4 Discussion

In August 2021, two families of Japanese rock ptarmigans were moved from their natural habitat in the Japanese Alps to two zoos for breeding. In the present study, we examined the OPG values of the parasites in the birds to clarify the dynamics of infection by two *Eimeria* species over several years. Surprisingly, oocysts were frequently detected in feces throughout the 2-year study, although the detections sometimes became negative. Generally, after the inoculation of matured oocysts, oocysts can be found in feces for a certain period of time that is referred to as the patent period. The patent period is species-specific, *e.g.,* it is 4–10 days for *E. ninakohlyakimovae* in goats (Vieira et al., 1997), 7–10 days for *E. colchici* in pheasants (Goldová et al., 2000), and 14–20 days for *E. macusaniensis* in guanacos and alpacas (Jarvinen, 2008). After the patent period, the oocysts are no longer detected in feces (Ford et al., 2001; Cheng et al., 2024). The results of the present study suggested that Japanese rock ptarmigans might be continuously infected with *Eimeria* species; however, we cannot completely rule out the possibility that the birds may have been repeatedly inoculated by oocysts in the zoos despite hygienic management efforts. *Eimeria* species of Japanese rock ptarmigans may remain dormant in the intestinal crypts or mucosa and initiate new merogonies intermittently, or possess the extra-intestinal developmental cycle like *Eimeria reichnowi* Yakinoff and Matschoulsky, 1935 and *Eimeria gruis* Yakimoff and Matschoulsky, 1935 of cranes (Carpenter et al., 1980), or *Isospora serini* Aragão, 1933 of canaries (Box, 1975). If *Eimeria* species of Japanese rock ptarmigans have these characteristics, they might be effective survival strategies of the oocysts in the cold environment that makes external sporulation impossible, and however, the clear evidence is not available to date. It is currently thought that after the initial infection by *Eimeria* species, the hosts gain acquired immunity against reinfection by the *Eimeria* species (Hashimoto et al., 2014). Indeed, in studies of experimental infections in Svalbard rock ptarmigans, infection by a low dose of oocysts isolated from Japanese rock ptarmigans appeared to induce acquired immunity that reduced the pathogenicity of future *Eimeria* infections (Matsubayashi et al., 2023). Although the immune mechanism of Japanese rock ptarmigans against *Eimeria* infections remains unclear, the contribution of acquired immunity in preventing the development of lower numbers of oocysts in the intestine might be low.

Among the examined female birds, the OPG value increased rapidly around only the time of hatching in birds that laid the eggs (Table 1, Table 2). The OPG value did not increase in female birds that were not paired for breeding or had unsuccessful breeding. Although the reason for this difference has not been clarified, it might be associated with some factors, *e.g.,* changes in endocrine hormones due to breeding or in immune status due to the burden and stress for brooding of eggs and caring for chicks. Interestingly, an increase in the OPG of female parents may increase the chance of transmission to chicks after they hatch, especially considering the coprophagic habit of chicks to eat the feces of their parents (Kobayashi et al., 2019). Although details on the pathogenicity of *Eimeria* species in chicks remain to be elucidated, it is possible that antibodies in the egg-yolk from parent birds, indigenous intestinal microflora specific to Japanese rock ptarmigans, and/or feeding on alpine plants (*Empetrum nigrum* var. *japonicum* or *Loiseleuria procumbens* etc.) possessing anticoccidial activity might reduce the pathogenicity of the parasites (Ushida et al., 2016; Kobayashi et al., 2020; Haraguchi et al., 2024). In the present study, the chicks at Nagano Chausuyama Zoo (two chicks of CH-Pa-F) died after shedding high levels of oocysts (>100,000 OPG); although the causes of death could not be determined, we speculate that the balance of factors in the intestine might be critical for the survival of the birds, and it may need to be considered in *ex situ* conservation and future re-introduction plans for Japanese rock ptarmigans.

The population of Japanese rock ptarmigans is thought to have formed during the ice age (Nakamura, 2007). Since then, they have developed intestines with a specific environment that is dependent on the intestinal flora, feeds, and protozoan parasites. As such, it is possible that the birds may coexist or have a symbiotic relationship with *Eimeria* parasites, *i.e.,* the parasites may be necessary for chicks to survive after they hatch. However, it is undeniable that infections by *Eimeria* species also negatively affect the health of the hosts. Further studies are required to clarify the possible symbiotic relationship between the birds and *Eimeria* parasites via changes in the intestinal environment.

## CRediT authorship contribution statement

**Nami Masakane:** Writing – original draft, Methodology, Investigation, Formal analysis, Data curation, Conceptualization. **Mei Harafuji:** Resources. **Yuki Arakawa:** Resources. **Tatsuhiko Yamakami:** Resources. **Naoya Tamura:** Resources. **Sayaka Tsuchida:** Resources, Methodology, Conceptualization. **Atsushi Kobayashi:** Resources, Methodology, Conceptualization. **Tomoyuki Shibahara:** Writing – review & editing, Methodology, Investigation. **Hiroshi Nakamura:** Resources, Project administration, Methodology, Investigation, Conceptualization. **Kazumi Sasai:** Methodology, Investigation, Funding acquisition, Conceptualization. **Kazunari Ushida:** Resources, Methodology, Investigation, Funding acquisition, Formal analysis, Data curation. **Makoto Matsubayashi:** Writing – review & editing, Writing – original draft, Project administration, Methodology, Investigation, Funding acquisition, Formal analysis, Data curation, Conceptualization.

## Ethics statement

In this study, ethical approval for animal experimentation was not necessary since all experiments were carried out without sacrificing any live animals. All examinations performed in the field study were permitted by the Ministry of the Environment of Japan. Fecal collection was performed in a non-invasive manner. No human participants were involved in the study.

## Declaration of competing interest

All the authors confirm that we declare that they have no conflict of interest.
